# Reliability assessment of a mechanism-based approach to post-injury knee magnetic resonance imaging interpretation by general radiologists

**DOI:** 10.4102/sajr.v22i1.1253

**Published:** 2018-06-04

**Authors:** James Stutterheim, Matthew D. Goodier

**Affiliations:** 1 Department of Radiology, University of Kwa-Zulu Natal, South Africa; 2 Department of Radiology, Grey’s Hospital, South Africa

## Abstract

**Background:**

A mechanism-based approach to post-injury knee magnetic resonance imaging (MRI) interpretation, following acute complex knee injury, is cited by several authors to provide increased reporting accuracy and efficiency, by allowing accurate prediction of injury to at-risk structures. This remains to our knowledge untested in a developing world setting and is of interest to us as South African general radiologists.

**Objective:**

To assess the reliability of a mechanism-based approach to complex post-trauma knee MRI interpretation when implemented by general radiologists in a South African setting, and compare our results with the findings of North American authors who compiled and assessed the same classification. To measure the agreement between the observers.

**Methods:**

A quantitative, observational, investigative, retrospective study was performed using a sample of 50 post-trauma knee MRI studies conducted at Grey’s Hospital, Pietermaritzburg. Two investigators independently applied the consolidated mechanism-based approach compiled by Hayes et al. as a research tool to interpret the knee MRI studies, blinded to each other’s findings.

**Results:**

Injury mechanism was assigned in 32% of cases by the principle investigator and in 20% of cases by the supervisor, with fair agreement between the observers (k = 0.39). The investigators agreed that 62% of cases were not classifiable by mechanism, 26% because of highly complex injury and 26% because of non-specific findings.

**Conclusion:**

Our findings indicate that the Hayes et al. classification is a non-ideal tool when used by general radiologists in our setting, as the pure injury mechanisms described in the classification were rare in our study group. Patient epidemiology and investigator experience are highlighted as potential limiting factors in this study. Despite this, we advocate that the concept of a mechanism-based approach for the interpretation of acute post-trauma knee MRI holds value for general radiologists, particularly in patients imaged before resolution of bone bruising (within 12–16 weeks of injury), and those injured in sporting and similar athletic activities.

## Background

A mechanism-based approach to radiological imaging interpretation, following complex musculoskeletal injury, has logical and useful clinical application because of the predictable synergisms that may occur within a number of complex joints. Several classifications have been developed on this basis, such as the Young–Burgess classification for pelvic fractures and Lauge–Hansen classification for ankle fractures.^1,2^ The knee lends itself favourably to this concept, firstly because of the complex interrelation of its primary and secondary stabilising structures, and secondly because of the high positive and negative predictive values of magnetic resonance imaging (MRI) for the diagnosis of injury to a wide range of soft tissue structures in the knee.^3,4,5^

It is well established that knee injuries occur commonly in the athletically active population, with the incidence of significant knee injury estimated to lie in the region of 500 cases per year per 400 000 population in a United Kingdom study.^6^ In a 10-year-long Swiss-based study of the epidemiology of knee injuries in over 17 000 athletically active patients, 50% of knee injuries were found to occur in patients between the ages of 20 and 29 years.^7^

Clinical examination is highly sensitive for diagnosing soft tissue injury in patients presenting, following knee injury such as occurring by falling, twisting injury or direct impact, with accuracy ranging between 75% and 96% for the diagnosis of significant ligament or meniscal injury demonstrated by Rayan et al.^8^ Magnetic resonance imaging is commonly used by the orthopaedic surgeon as an adjunct to clinical examination and is most useful in the setting of equivocal clinical examination or acute complex knee injury, when the presence of swelling and pain may limit the accuracy of clinical examination.^4,8,9^ Magnetic resonance imaging is highly accurate in the diagnosis of internal derangements of the knee.^10^ A systematic review comparing MRI and arthroscopy findings found high sensitivity and specificity for MRI detection of meniscal and cruciate ligament injury with figures between 88% and 99% for all structures except for a relatively lower sensitivity of 79% for the detection of lateral collateral ligament injury.^10^

A mechanism-based approach to complex post-trauma knee MRI interpretation is cited by several authors in the recent literature to provide increased reporting accuracy and efficiency, by allowing accurate prediction of injury to at-risk structures.^3,4,11,12^ An understanding of the biomechanics of the knee can heighten awareness of injury to key stabilising structures such as the posterolateral or posteromedial corner, as well as subtle but important injuries to key structures such as the anterior cruciate ligament (ACL) or posterior cruciate ligament (PCL). Undiagnosed injuries left untreated could lead to chronic instability and eventual failure of surgical repair of structures such as the ACL.^3^

Such an approach is complementary to an initial thorough screening of individual structures, using an anatomical approach, as detailed in comprehensive musculoskeletal imaging texts such as Stoller’s ‘Magnetic Resonance Imaging in Orthopaedics and Sports Medicine’.^13^ The radiologist requires a clear appreciation of the spectrum of injury types and grades for each structure of the knee, including partial and full thickness ligament tears, the wide variety of meniscal tears, and the complex anatomy of and appearance of injury at the posterolateral corner.^14^

We, as general radiologists seeking to raise reporting standards by following emerging international trends, took interest in the clinical benefits accredited to a mechanism-based approach in post-trauma MRI knee interpretation, notably because of its proposed increased accuracy and efficiency.^4^ To our knowledge, such an approach has not been tested in a developing world setting where epidemiology of knee injury differs, and patient presentation and referral may be delayed.

The consolidated mechanism-based pattern approach for the interpretation of acute post-trauma knee MRI compiled by Hayes et al.^3^ is the most comprehensive approach of its kind in the recently published literature, with similar articles on this topic taking a more descriptive approach. The utility of a mechanism-based imaging approach is directly proportional to the percentage of cases that can be thereby classified – the Hayes et al. classification stands out in this regard, with 85% of cases classifiable by the approach in their setting.

This classification comprises 10 common injury mechanisms and emphasises the initial identification of the pattern of bone bruising, which in combination with the identification of ligamentous and capsular injury allows one to identify the pattern of the injury mechanism. A key component of the classification is the differentiation of impaction and avulsion bone bruising: both are maximal in the subcortical region of the trauma force; however, impaction bruising tends to be larger, has variable location and, when due to impaction across the joint, is seen at contiguous sites on either side of the joint; avulsion oedema, however, is typically more confined and localised at the precise attachment site of the ligament or tendon injured by traction force.^14^ Although some studies suggest onset of resolution of bone bruising from as early as 7–12 weeks,^4^ and persistence as long as 42 weeks,^15^ a detailed study by Frobell has estimated mean resolution time for femoral bone bruising at 12 weeks and for tibial bone bruising at 24 weeks.^16^

The primary objective of this study is to quantitatively evaluate the reliability of a mechanism-based approach to post-trauma knee MRI interpretation when implemented by general radiologists in a South African setting, using the Hayes et al. classification.

## Methodology

A quantitative, observational, investigative, retrospective study was performed, using a sample of 50 post-trauma knee MRI studies conducted at Grey’s Hospital, Pietermaritzburg, a state-funded tertiary referral centre and teaching hospital in Pietermaritzburg, KwaZulu-Natal, South Africa, between 03 January 2012 and 24 June 2014.

Criteria for inclusion were adult patients (>12 years of age) who had undergone knee MRI investigation within 16 weeks of traumatic injury. Sixteen weeks was chosen as the most reasonable cut-off. This was firstly to ensure that the majority of cases would lie within the window of detection of bone bruising. Secondly, this was in light of the frequently delayed timing between injury and MRI in our setting (average time from injury to MRI for a chronological sample of 30 patients imaged at Grey’s Hospital was 9.5 months).

Normal studies and studies performed for non-traumatic knee pathology were excluded. Three of the initial 50 selected cases were eliminated (one normal study, one patient with imaging findings of septic arthritis and one with a bone tumour). The next three sequential cases with imaging findings of traumatic injury were added to restore a number of 50 cases. The study series consisted of a total of 50 knees imaged from 47 patients, as 3 patients had injuries to both knees.

All examinations were performed using the same 1.5 Tesla magnet MRI scanner (Phillips Medical Systems, South Africa). Patients were scanned using a dedicated knee coil and standard local protocol (sagittal T1W, sagittal STIR T2W, axial and coronal proton density with fat saturation, sagittal 3D SPIR sequences).

### Research tool

The mechanism-based classification system for complex knee injuries compiled by Hayes et al. was used as the research tool.^3^ The classification comprises 10 common injury mechanisms and their key individual injury components.

The authors devised an image-based quick reference summary of common knee injury mechanisms aimed to improve efficiency when using the Hayes et al. classification. The summary is based on the 10 common injury patterns described by Hayes et al. and includes for each mechanism a schematic diagram of the position of the knee at the time of injury (sketches were produced by the principal investigator to aid conceptualisation of the position of the knee at the time of injury for each mechanism), corresponding bone bruise and soft tissue injuries outlined on a normal MRI scan, and a brief description of the imaging findings. We found it a challenging task to continually cross-reference imaging findings with typed descriptions. The summary yields the classification more user-friendly, especially for those new to or not frequently reporting post-trauma knee MRI cases (refer to Online Appendix 1).

The most commonly encountered mechanism in the Hayes et al. study, nicknamed the ‘O’Donoghue’s unhappy triad’, may occur with either contact or non-contact force and occurs with valgus and internal rotation injury of the flexed knee. This injury results in a typical bone bruise pattern with associated ACL and medial collateral ligament (MCL) injury.

**FIGURE 1:**
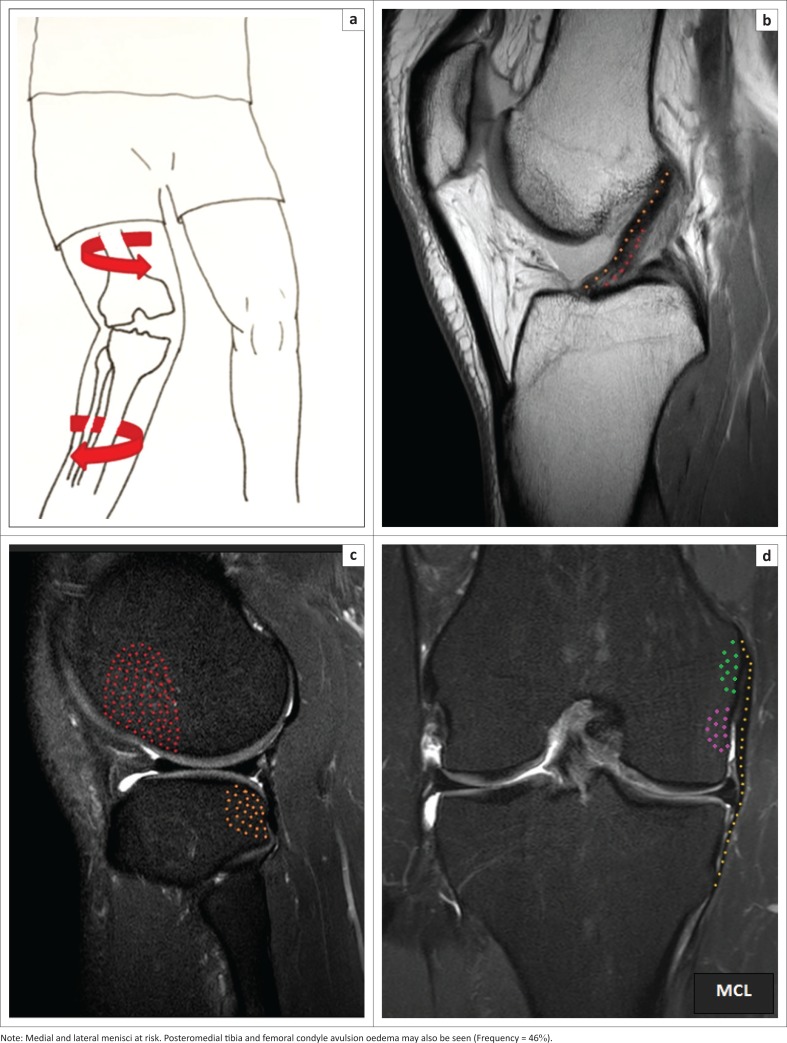
Flexion, valgus and external rotation: (a) Schematic depicts excessive sudden internal rotation of femur on ‘fixed’ tibia (effective external tibial pivot) with knee in flexed valgus position, (b) anterior cruciate ligament (ACL) tear that results from excessive rotation, (c) non-contiguous impactions at the lateral femoral condyle and posterolateral tibia that occur once the ACL has given way and (d) right knee medial collateral ligament (MCL) tear that is often incomplete; look for avulsion bone bruise at deep (purple) and superficial (green) MCL portion attachments.

For the sake of comparison, the digital tool made the classification more practical, as seen in Figure 1 extracted from quick reference guide classification^3^ (also see Online Appendix 1: Figure 6).

A retrospective review was performed on a sample of 50 digital MRI knee studies from a picture archive and communication system (PACS), by both the principal investigator (senior registrar at outset of study, now a radiologist in general private practice) and supervisor (radiologist now with 4 years experience in general state practice). The investigators descriptively recorded relevant imaging findings (i.e. bone bruising, ACL, PCL, MCL, lateral collateral ligament [LCL] and capsular injuries) relevant to the Hayes et al. mechanism-based classification, and remained blinded to each other’s findings, and to the injury circumstances during the imaging interpretation phase. Impaction and avulsion bone bruising were differentiated where applicable. The investigators independently correlated the MRI findings of each case with the 10 mechanisms of the Hayes et al. classification. Where there was a clear match between the imaging findings and a particular mechanism in the classification, an injury mechanism was assigned.

The investigators remained blinded to one another’s findings and to the injury circumstances so that a reflection of the performance of the classification could be established for each interpreter. The completed investigator findings were then compared to assess the number of cases that were classifiable or non-classifiable by mechanism according the Hayes et al. classification.

Where provided, knee injury circumstances were recorded from the MRI request form history, and injuries were classified into the categories motor vehicle and pedestrian vehicle accidents (MVA/PVA), falls, sporting injury and injury not specified. This was performed following completion of the imaging interpretation phase, to avoid biasing the assignability of a classification, as the focus of the study was assessment of the reliability of an injury mechanism classification based on imaging findings alone.

Full ethics approval was granted by the Biomedical Research Ethics Committee: University of KwaZulu-Natal Biomedical Research Ethics Committee with reference number BE518/14.

### Statistical analysis

Descriptive statistics were employed to determine the percentage of cases classifiable and non-classifiable by the investigators, as well as to determine the potential reasons for non-classifiability to quantitatively assess the reliability of the research tool.

The chi-squared test and *p*-value were used to determine whether there was a significant difference between the percentage of cases classifiable by the investigators and Hayes et al. The Fleiss’ kappa measure of agreement was used to calculate the interrater reliability. Use of Statistica was employed for statistical analysis.

## Results

Frequency distribution tables for gender, age, timing between injury and MRI, and knee injured are specified in Tables 1, 2, 3 and 4, respectively.

**TABLE 1:** Frequency distribution: Gender.

Gender	*N*	%
Female	19	38
Male	31	62
**Total**	**50**	**100**

**TABLE 2:** Frequency distribution: Age.

Age	*N*	%	Cumulative	
			*N*	%
14–19	7	14	7	14
20–29	13	26	20	40
30–39	19	38	39	78
40–49	7	14	46	92
50–59	3	6	49	98
60–69	1	2	50	100

**TABLE 3:** Frequencies: Injury to magnetic resonance imaging (months).

Injury to MRI	*N*	%	Cumulative	
			*N*	%
0.00–0.99	16	32	16	32
1.00–1.99	14	28	30	60
2.00–2.99	12	24	42	84
3.00–3.25	8	16	50	100

MRI, magnetic resonance imaging.

**TABLE 4:** Frequency distribution: Side injured.

Side injured	*N*	%
Left	28	56
Right	22	44
**Total**	**50**	**100**

### Assignability of mechanism

There was an agreement between both investigators on the assignable injury mechanism in 14% of cases. This was significantly less than the 85% classifiable cases achieved by Hayes et al. using the same classification (*p* < 0.0005; c^2^: *V* = 1.48; Table 5). Individually, injury mechanism was assigned in 32% by the principal investigator and in 20% by the supervisor. The investigators both agreed that a mechanism could not be assigned in 62% of cases. This was because of high injury complexity in 26%, non-specific findings in 26% and insufficient findings in 10% of the total cases. For the total 76% of assignability and non-assignability of a mechanism, there was fair agreement between the observers (Fleiss’ kappa coefficient *k* = 0.39), as shown in Table 6. There was disagreement on the presence of a classifiable injury mechanism in 24% of cases.

**TABLE 5:** Injury mechanism assignment.

Variable	No		Yes		Chi square (*df* = 1; *n* = 50)	*p*	Cramer’s *V* value
	*N*	%	*N*	%			
Principal investigator	34	68	16	32	110.16	<0.005	1.48
Supervisor	40	80	10	20	165.69	<0.005	1.82

Chi square, Goodness of fit test.

**TABLE 6:** Analysis of agreement for classifiable and non-classifiable mechanisms.

Inspected	Matched		Kappa	SE kappa	*z*-statistic	*p*	95% confidence interval
	*N*	%					
50	38	76	0.38	0.14	2.66	0.004	61.83–86.94

In the cases where there was disagreement on a classifiable injury mechanism, the two readers agreed that bone bruising was present in 7 of these 12 cases.

### Bone bruising

Bone bruising was present in 84% of cases overall. There was complete and near-complete agreement on the presence of bone bruising in 96% of cases (exact agreement on findings in 66% of total cases and near-complete agreement in 30% of total cases, with the latter arbitrarily defined by the authors as a minor variation in interpretation at a single site of bone bruising). Of these cases, 54% were agreed to be non-classifiable by mechanism; 13 of 27 cases (48%) were agreed to be non-classifiable because of non-specific findings and 12 of 27 cases (44%) were agreed to be non-classifiable because of high injury complexity. Two cases were not classifiable because of insufficient findings. There was disagreement on bone bruising in two cases (4%), meaning that the investigator interpretation of bone bruising sites differed significantly. There was no bone bruising present in 14% of cases, and there was agreement on non-assignability in all of these cases.

### Injury circumstances

Thirty-four per cent of injuries were sustained during MVA/PVA, 24% in sporting and similar athletic activities and 20% in falls. In 22% of cases, injury circumstances were not provided. Of PVA/MVA injuries, 2 of 17 knees had classifiable mechanisms, and seven could not be classified because of high injury complexity. Of sports and related injuries, 2 of 12 knees had classifiable injuries, four could not be classified because of indeterminate findings, 3 could not be classified because of insufficient findings and there was disagreement on mechanism in 3 knees.

There were insufficient imaging findings in 10% of cases, with absent bone bruising in four out of these five cases (Note: above findings according to both observers).

## Discussion

The major finding of this study is the low rate of cases (14%) agreed classifiable by mechanism, compared to 85% in the original Hayes et al. study. Pure injury mechanisms outlined in the Hayes et al. classification were rare in our study group, and reasons for this are explored below.

There was disagreement on injury mechanism in 12 cases (24% of the study sample). Of these, in seven cases there was agreement between the observers on the sites of bone bruising. Most of these cases had extensive bruising at multiple sites, indicating complex mechanism not fitting with a typical bone bruising pattern from the Hayes et al. classification. There was disagreement on the significance of soft tissue injury between the investigators in these cases. In several cases, one investigator assigned a mechanism within a more complex injury, while the other did not. In no single case was a different mechanism assigned by both investigators. Hayes et al. do not specify their exact methodology in the application of their classification. It may be that each individual case requires careful scrutiny to identify synergistic injury components possibly within a more complex injury, as well as an awareness of variances that may occur with each mechanism. Experience may strongly affect the ability to accurately achieve this.

Clearer guidelines on the implementation of a mechanism-based classification are anticipated to reduce subjective variability between observers. Examples might include minimum criteria, exclusion criteria and a points system for identifying mechanisms.

Bone bruising is the fundamental starting point in the identification of knee injury mechanism on MRI. The finding of high combined complete and near-complete agreement regarding the presence or absence of, as well as the sites of, bone bruising in 96% of cases, with bone bruising agreed present in 84% of cases overall, reinforces that bone bruising is a reliable radiological finding. Despite this, there was a low incidence of the typical bone bruise patterns presented in the Hayes et al. classification, which are fundamental to a mechanism-based classification. A corresponding high percentage of cases with agreement on bone bruising, namely 54% could not be classified by mechanism, predominantly because of indeterminate findings and high injury complexity. MacMahon and Palmer^4^ discuss countless circumstances that lead to traumatic knee injury, involving numerous combinations of the 12 possible movements at the knee and occurring by contact and non-contact forces. Questions are thus posed as to whether the Hayes et al. classification (or any similar classification) can adequately encompass the full range of common knee injuries, and whether pure mechanisms occur less commonly than they are postulated to. Additional injury components such as medial or lateral tibial translation, discussed by MacMahon and Palmer in one of their published cases, add another dimension to the topic and are not included in the Hayes et al. classification.

Soft tissue injury is complimentary to the detection of fundamental injury mechanism. Typical soft tissue injuries were identified jointly by both investigators in only the 14% of cases classified by both investigators. By definition injury mechanism cannot be assigned in the absence of a typical bone bruise pattern.

The high incidence and agreement on the presence and site of bone bruising suggests that the inclusion criterion of cases imaged within 4 months of injury was adequate. However, despite this, MacMahon and Palmer^4^ indicate that bone bruising is postulated by some authors to resolve by 6–12 weeks. In light of this, a further study could look at applying a mechanism-based image interpretation approach within this window to ensure that subtle bone contusions will not be missed.

There was a high combined percentage of MVA/PVA injuries (34%) and falls (24%) of over 50% in our study sample. The 31% MVA/PVA statistic correlates with the generally high South African road accident morbidity and mortality reflected by the average 35.8 deaths per 100 000 population, which is almost double world averages of 19 deaths per 100 000 in 1999.^17,18^ Patients injured by MVA/PVA or falls from a height are more likely to have had complex soft tissue injuries because of high velocity, often with accompanying fractures, and it may be expected that in such cases a single injury mechanism will generally not apply. In this study, of the 31 cases agreed non-categorisable, 17 were because of MVA/PVA and falls combined. This suggests that the classification was less reliable in this subset of patients.

A high percentage of classifiable cases in the Hayes et al. study sample (46%) were because of rotational ‘pivot-shift’ injury (classical non-contact rotational injury seen commonly in sports such as soccer and skiing),^4^ whereas only one case of a pivot-shift mechanism was agreed present by the observers in our study sample. The relatively low 24% of sport-related injuries in our setting and high number of combined MVA/PVA and falls, suggests considerably different injury epidemiology between our study group and the initial Hayes et al. study population. It may be suggested that the Hayes et al. classification is best applied in the setting of sporting and recreational athletic activities, when lower velocity trauma and pure injury mechanisms will be more likely. It was surprising to note that only 2 of 12 sports injuries were classifiable. Although this is lower than anticipated, it may be argued that there are too few sports-related injuries in our study sample to draw conclusion from. A further study focusing on a South African study sample with sports injuries would be beneficial in confirming this.

The South African radiology environment is, following global trends, in evolution from generalist to sub-specialist structure; however, the predominant need within the system remains general radiologists, in light of the high burden of disease and its resource-constrained health services. The authors, both general radiologists, recognise the skills gap that will exist between general radiologists, especially those less experienced, and sub-specialist trained musculoskeletal radiologists dedicated to this field in their every-day practice. This lack of subspecialty training and experience could have been at least partly responsible for the fewer cases classifiable by mechanism in the study group, as well as there being only fair agreement between the observers. Accurate and consistent differentiation between impaction and avulsion bone marrow patterns at varying sites, fundamental to the Hayes et al. classification, is a skill that will develop with experience and practice.

### Limitations

The sample size of 50 cases may be considered a limitation compared with the 100 cases in the Hayes et al. study.

This study assessed the reliability of a mechanism-based classification when applied by general radiologists who were both relatively inexperienced. Further studies assessing the performance of the classification system amongst experienced general radiologists and/or subspecialty musculoskeletal radiologists would be of interest.

## Conclusion

Is the application of a mechanism-based approach for the interpretation of acute post-trauma knee MRI appropriate for general radiologists? We believe yes, as such an approach proposes significant clinical benefits and aims to raise reporting standards and quality. Is the Hayes et al. classification in particular, a useful tool to achieve this? We have shown that this is a non-ideal tool when used by general radiologists in our setting, in essence because pure injury mechanisms were rare. Despite this, we propose that following a thorough initial assessment of individual structures within the knee, a global search for common injury patterns is still warranted, based primarily on bone bruising. This is likely particularly beneficial in patients injured during sporting and similar athletic activities, and within the period of maximal visibility of bone bruising, ideally within 12 weeks of injury. Perhaps the identification of synergistic components of complex knee injuries is more clinically relevant than limiting this concept to pure injury mechanisms only. Further research is anticipated to identify the effect of investigator experience on the reliability of MRI interpretation with a mechanism-based approach. A higher skill and experience level may be required to accurately identify synergistic injury components in the setting of highly complex injuries.
